# Microscopic Phase-Field Modeling with Accurate Interface Thickness Representation: Applied to Ceramic Matrix Composites

**DOI:** 10.3390/ma18194496

**Published:** 2025-09-27

**Authors:** Tong Wang, Xiaofei Hu, Zhi Sun, Weian Yao

**Affiliations:** 1 State Key Laboratory of Structural Analysis, Optimization and CAE Software for Industrial Equipment, Dalian University of Technology, Dalian 116024, China; hardysun0107@163.com (T.W.); zhisun@dlut.edu.cn (Z.S.); ywa@dlut.edu.cn (W.Y.); 2 International Center for Computational Mechanics, Dalian University of Technology, Dalian 116024, China; 3 State Key Laboratory of Intelligent Construction and Healthy Operation and Maintenance of Deep Underground Engineering, China University of Mining & Technology, Xuzhou 221116, China; 4 Yunlong Lake Laboratory of Deep Underground Science and Engineering, China University of Mining & Technology, Xuzhou 221116, China

**Keywords:** ceramic-matrix composites (CMCs), interface, fracture, phase field model

## Abstract

Ceramic matrix composites (CMCs) are promising candidates for high-temperature structural applications. However, their fracture toughness remains low due to strong chemical bonding between fibers and the matrix. To improve toughness, engineered interfaces such as pyrolytic carbon (PyC) and hexagonal boron nitride (h-BN) are commonly introduced. These interfaces promote crack deflection and fiber bridging, leading to improved damage tolerance and pseudo-ductile behavior. To investigate the influence of interface thickness on mechanical performance and to identify optimal thickness ranges, we propose a microscopic phase-field model that accurately resolves interface thickness and material contrast. This model overcomes the limitations of conventional smeared interface approaches, particularly in systems with variable interface thickness and closely packed fibers. We apply the model to simulate the fracture behavior of unidirectional SiC fiber reinforced SiC matrix (SiC_f_/SiC_m_) composites with PyC and h-BN interfaces of varying thickness. The numerical results show strong agreement with experimental findings from the literature and reveal optimal interface thicknesses that maximize toughening effects. These results demonstrate the model’s predictive capabilities and its potential as a tool for interface design in brittle composite systems.

## 1. Introduction

Fiber-reinforced ceramic matrix composites (CMCs) have gained widespread attention for advanced thermal-structural applications due to their high-temperature strength, oxidation resistance, and damage tolerance [1,2]. Their low density and excellent thermomechanical performance make them promising replacements for traditional superalloys in aerospace and energy systems [3], where weight reduction and efficiency are critical. As operating temperatures continue to rise in gas turbines [4], nuclear reactors, and hypersonic platforms, CMCs are increasingly indispensable for components exposed to extreme thermal and mechanical loads [5]. Among various CMCs, silicon carbide fiber-reinforced silicon carbide matrix composites (SiC_f_/SiC_m_) stand out for their chemical compatibility, low thermal expansion mismatch, and stability in oxidizing environments [6]. They are already used in non-load-bearing components such as combustor liners and insulation parts, and with advances in processing and interface design, their application is expanding toward high-load components like turbine vanes and blades [7]. This trend is driven by the need to improve thermal efficiency, extend service life, and reduce weight—especially in environments where metal alloys suffer from oxidation or creep. Despite their advantages, SiC_f_/SiC_m_ composites still face challenges related to fracture toughness, which is highly sensitive to microscale fiber–matrix interactions. Strong chemical bonding formed during high-temperature sintering, while beneficial for load transfer, often inhibits key toughening mechanisms such as crack deflection and fiber pull-out—resulting in brittle fracture behavior [8,9,10].

**Figure 1:**
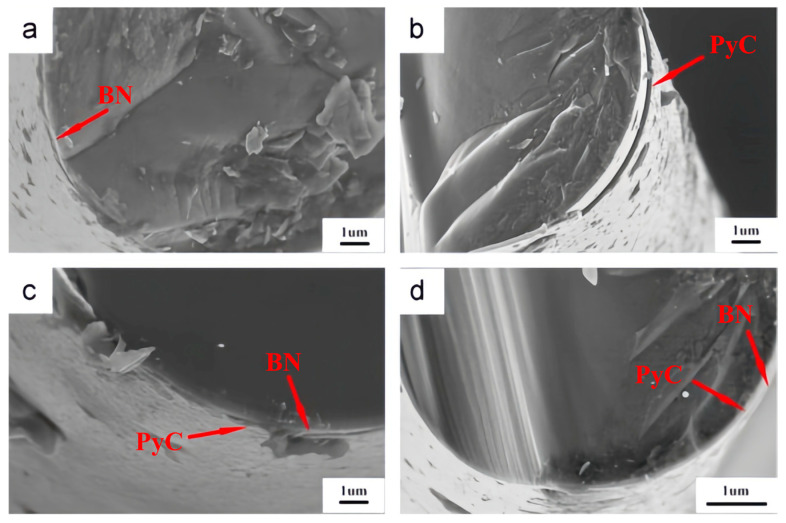
Fracture surface morphology of SiC fibers with different coatings: (**a**) dip-coated BN, (**b**) PyC, (**c**) dip-coated BN/PyC, and (**d**) PyC/dip-coated BN [14].

To address this, engineered interfaces such as pyrolytic carbon (PyC) and hexagonal boron nitride (h-BN) are commonly introduced between fibers and the matrix [11,12], as shown in Figure 1. These weak interfaces facilitate crack deflection and fiber bridging during fracture, which in turn improves damage tolerance and enables pseudo-ductile response [13]. In particular, PyC interfaces have been shown to significantly enhance toughness and structural reliability in SiC_f_/SiC_m_ composites. The mechanical performance of these systems is closely tied to interface thickness. Therefore, when studying the mechanical response of ceramic matrix composites, the influence of the interface thickness must be taken into account.

The mechanical behavior of ceramic matrix composites (CMCs) is commonly investigated using both experimental techniques and numerical simulations. While experimental methods [15,16]—such as mechanical testing, microscopy, and fractography—offer direct insight into damage and failure mechanisms, they often involve high material costs, complex sample preparation, and limited flexibility in controlling microstructural parameters. This makes it challenging to systematically study the influence of interfacial and structural variations on composite performance. In contrast, numerical simulation provides a powerful and efficient alternative for exploring damage evolution and fracture processes under controlled conditions. It enables detailed analysis of material response at multiple scales and offers predictive capabilities that complement experimental findings. As such, the development of robust and adaptable numerical approaches is essential for advancing the design and optimization of CMCs.

Several computational methods have been established for simulating fractures in composite materials, including the cohesive zone model (CZM) [17,18,19], extended finite element method (XFEM) [20,21,22], virtual crack closure technique (VCCT) [23,24], and peridynamics [25,26]. While effective in certain applications, these methods often face limitations such as mesh dependency, predefined crack paths, or poor adaptability to complex microstructures. The phase field method for fracture has emerged as a promising alternative, particularly well-suited for modeling complex crack initiation and propagation in brittle [27,28,29,30,31,32,33,34,35] and multi-phase materials [36,37,38]. By introducing a continuous scalar field to represent crack evolution [39,40,41,42], the phase-field approach eliminates the need for explicit crack tracking and can naturally capture arbitrary crack paths, coalescence, and branching. Its variational foundation ensures energy consistency and facilitates coupling with other physical fields, making it highly versatile for simulating fractures in CMCs. Given the inherently brittle nature of CMCs and the critical role of fiber–matrix interactions, this study employs a phase field modeling framework to accurately simulate damage initiation and growth in ceramic composite microstructures.

In phase-field modeling of fracture in composite microstructures, two main approaches are currently used to represent the fiber–matrix interface. The first approach explicitly defines the interface as a separate region within the model geometry [43,44,45]. This method directly assigns thickness and material properties to the interface, but requires generating a separate mesh for each interface configuration. It also demands fine mesh resolution across the interface, especially for thin layers, resulting in high computational cost and modeling complexity. The second approach is the smeared interface method [46,47,48,49], which uses a continuous transition function to gradually interpolate material properties. This avoids the need to explicitly define the interface geometry. Due to its flexibility and efficiency, this method has gained increasing interest and is considered a promising direction for large-scale or parametric simulations. However, current smeared interface methods often assume thin interface characteristics and struggle to capture variations in interface thickness, especially in microstructures with closely spaced fibers. As a result, their accuracy in predicting mechanical response is limited.

To overcome the limitations, this work proposes a novel unified framework for smeared interface modeling in phase-field model of microscopic fracture. Without explicit geometric partitioning of the interface region in the model, the proposed approach avoids the need to remesh or remodel for each interface thickness, significantly improving computational efficiency. The method is demonstrated in this study through two common applications in ceramic matrix composites. First, it is used to investigate the effect of varying h-BN interface thickness in a single-fiber SiC_f_/SiC_m_ composite under transverse tension. Second, it is applied to simulate the three-point bending fracture behavior of PyC-coated SiC_f_/SiC_m_ composites, where the predictions are validated against experimental results. These examples highlight the method’s accuracy in capturing interface-driven fracture mechanisms and its potential for guiding interface design in complex composite microstructures.

## 2. Conventional Phase-Field Modeling in Microscopic Composite Models: Methods and Limitations

In micromechanical modeling of composite materials, the microstructure is typically considered as a heterogeneous system composed of fibers, matrix, voids, and the fiber–matrix interface [36,50,51]. Although the interface plays a critical role in mechanical behavior—particularly in damage initiation and failure evolution—its extremely small thickness often prevents accurate measurement of geometric and material properties. This poses significant challenges for phase-field modeling of failure processes. To address this issue, the auxiliary interface phase field approach has been developed, which constructs an equivalent continuous material field to represent the fiber, matrix, and interface within a unified computational framework. However, conventional smearing functions used to interpolate material properties across phases exhibit notable limitations when the thickness of the interface needs to be taken into consideration or when fiber spacing is small. In such cases, the overlap of smeared regions leads to significant numerical errors.

### 2.1. Overview of Phase-Field Theory for Fracture

**Figure 2:**
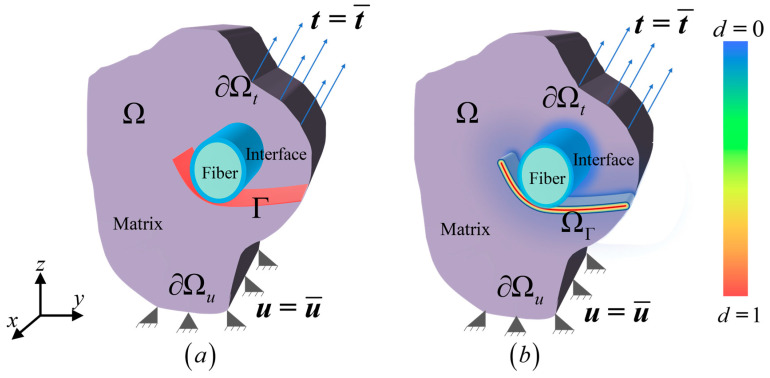
Diagram of a three-dimensional system containing (**a**) discrete cracks and (**b**) smeared cracks.

For a micro-scale structure Ω containing crack set Γ shown in Figure 2, it is considered to be consisted of matrix domain $\Omega_{m}$, fiber domain $\Omega_{f}$ and interface domain $\Omega_{i}$. The traction $\bar{t}$ is applied on the boundary $\partial \Omega_{t}$ and the displacement $\bar{u}$ is prescribed on the boundary $\partial \Omega_{u}$. According to the Francfort-Marigo variational principle [41], the total potential energy of the system W is

(1) $$\begin{array}{ll} W & =W_{m}+W_{f}+W_{i}+W_{d}-W_{ext}\\ & =\int_{\Omega_{m}-\Gamma }\Phi_{\varepsilon }dV+\int_{\Omega_{f}-\Gamma }\Phi_{\varepsilon }dV+\int_{\Omega_{i}-\Gamma }\Phi_{\varepsilon }dV+\int_{\Gamma }G_{c}dS-\int_{\partial \Omega_{t}}t\cdot u\;dS \end{array}$$

where $W_{ext}$ is the total potential of the external force. $W_{m}$, $W_{f}$ and $W_{i}$ are elastic potential of the matrix, fiber and interface zones, $W_{d}$ is the fracture energy, $\Phi_{\varepsilon }$ is the strain energy density in undamaged region, $G_{c}$ is the critical energy release rate.

The phase-field model, as a smeared method, allows the crack set Γ to be approximately described by a continuous scalar phase-field variable $d\in \left[0,\;1\right]$, as shown in Figure 2b. The material is intact when d=0, and fully failed when d=1. Thus, Equation (1) can be rewritten as [42,52]

(2) $$W=\int_{\Omega }\omega \left(d\right)\Phi_{\varepsilon }dV+\int_{\Omega }G_{c}\gamma \left(d,\nabla d\right)dV-\int_{\partial \Omega_{t}}t\cdot u\;dS$$

where $\omega \left(d\right)$ is the energy degradation function, $\gamma \left(d,\nabla d\right)$ is the crack surface density functional defined as [53]

(3) $$\gamma \left(d,\nabla d\right)=\frac{1}{c_{0}}\left[\frac{\alpha \left(d\right)}{l_{0}}+l_{0}{\left|\nabla d\right|}^{2}\right]$$

where $l_{0}$ is a scalar internal length scale parameter used to control the width of the regularization area, $\alpha \left(d\right)$ is the crack geometric function which characterizes homogenous evolution of phase field, $c_{0}=4\int_{0}^{1}\sqrt{\alpha \left(x\right)}\;dx$ is a scaling parameter ensuring the regularized crack surface approximates the discrete crack surface Γ.

The AT2 model [39] and the unified phase field model proposed by Wu [53]—also referred to as the phase field regularized cohesive zone model (PF-CZM)—are among the most widely used approaches in fracture phase field modeling. The specific functional forms and corresponding parameters of these models are summarized in Table 1. Notably, PF-CZM can represent various cohesive zone models by tuning its parameters, and two commonly adopted types are presented in the table.

In order to ensure that cracking only takes place under tensile stress state, the elastic potential is split into tensile part $\Phi_{\varepsilon }^{+}$ and compressive part $\Phi_{\varepsilon }^{-}$, and only the tensile part contributes to the evolution of the phase field. Then, Equation (2) is changed into:

(4) $$W=\int_{\Omega }\omega \left(d\right)\Phi^{+}+\Phi^{-}dV+\int_{\Omega }G_{c}\gamma \left(d,\nabla d\right)dV-\int_{\partial \Omega_{t}}t\cdot u\;dS$$

In this paper, two methods are employed for the elastic potential decomposition. The first method is based on principal strains [54]:

(5) $$\Phi^{\pm }=\frac{1}{2}\lambda {\langle \mathrm{tr}\left(\hat{\varepsilon }\right)\rangle }_{\pm }^{2}+\mu \mathrm{tr}\left({\langle \hat{\varepsilon }\rangle }_{\pm }^{2}\right)$$

where λ and μ are the Lamé constants. ${\langle x\rangle }_{\pm }:=\left(x\pm \left|x\right|\right)/2$ is the Macaulay bracket. $\hat{\varepsilon }$ is the principal strain tensor. The second method is based on principal stresses [46]:

(6) $$\Phi^{\pm }=\frac{1+v}{2E}\mathrm{tr}\left({\langle \hat{\sigma }\rangle }_{\pm }^{2}\right)-\frac{v}{2E}{\langle \mathrm{tr}\left(\hat{\sigma }\right)\rangle }_{\pm }^{2}$$

where v is Poisson’s ratio. $\hat{\sigma }$ is the principal stress tensor. Meanwhile, In order to ensure that the damage does not heal itself, i.e., the requirement of $\dot{d}\geq 0$, Miehe et al. [55] introduced a historically relevant variable:

(7) $$H=\underset{\tau \in [0,t]}{\max}\left\{\Phi^{+}\left(\varepsilon \left(x,\tau \right)\right)\right\}$$

The variation in Equation (4) can be given as follows:

(8) $$\begin{array}{ll} \delta W & =\int_{\Omega }\left(\omega \left(d\right)\frac{\partial {\bar{\Phi }}^{+}}{\partial \varepsilon }:\delta \varepsilon +\frac{\partial {\bar{\Phi }}^{-}}{\partial \varepsilon }:\delta \varepsilon \right)dV\\ & \;+\int_{\Omega }\omega^{\prime }\left(d\right){\bar{\Phi }}^{+}\delta ddV+\int_{\Omega }\frac{G_{c}}{c_{0}}\left[\frac{\alpha^{\prime }\left(d\right)}{l_{0}}\delta d+2l_{0}\nabla d\nabla \delta d\right]dV\\ & \;-\int_{\partial \Omega_{t}}t\cdot \delta udS-\int_{\Omega }f\cdot \delta udV \end{array}$$

Then the governing equations and boundary conditions for the current problem can be obtained as:

(9) $$\left\{\begin{array}{cc} \nabla \cdot \left[\omega \left(d\right)\sigma^{+}\right]+\nabla \cdot \sigma^{-}+f=0 & \;\;\;\left(x,y\right)\in \Omega \\ \omega^{\prime }\left(d\right)H+\frac{G_{c}}{c_{0}}\left[\frac{\alpha^{\prime }\left(d\right)}{l_{0}}-2l_{0}\Delta d\right]=0 & \;\left(x,y\right)\in \Omega \\ \left[\omega \left(d\right)\sigma^{+}+\sigma^{-}\right]\cdot n\;=\;t & \;\;\left(x,y\right)\in \partial \Omega_{\;t}\\ \nabla d\cdot n=0 & \;\;\left(x,y\right)\in \partial \Omega \\ \;u\;=\;\bar{u} & \;\;\left(x,y\right)\in \partial \Omega_{u} \end{array}\right.$$

The above represents a coupled system consisting of the modified stress equilibrium, given by the first equation, and the phase-field evolution, described by the second equation. The third and fifth equations correspond to the natural and essential boundary conditions associated with the stress equilibrium, while the fourth equation provides the natural boundary condition related to the phase-field evolution. Although the satisfaction of the fourth equation may appear trivial, it in fact imposes an upper bound on the choice of the regularization parameter $l_{0}$ with respect to the domain size, ensuring that the phase-field sufficiently decays to zero at the boundary.

### 2.2. An Auxiliary Phase Field for Fiber-Matrix Interface

**Figure 3:**
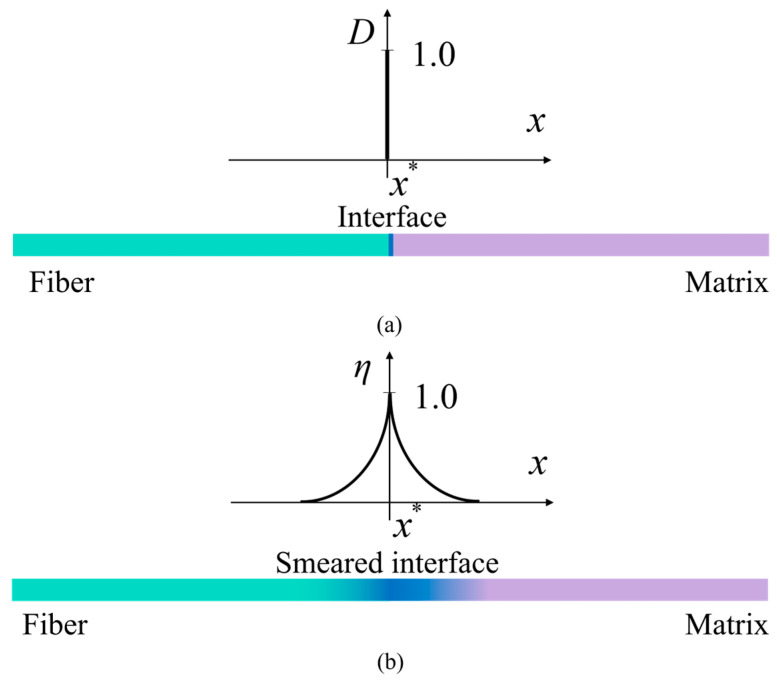
(**a**) Discrete representation and (**b**) smeared representation of the interface in a one-dimensional bar.

An auxiliary interface phase field is introduced to generate an equivalent material field representing the three material phases. For a better illustration of the regularization of the interface material using phase field model, a one-dimensional long bar is used to explain the theory [37]. Let us consider the bar with volume $\Omega =\Gamma_{L}\times L$ in Figure 3a, where $\Gamma_{L}$ is the cross-sectional area, and L is the length of the bar. This bar is made up of three materials. The cross-section area of the bar is a unit, and an interface $\Gamma_{i}$ locates at $x=x^{*}$. In this case, the interface can be represented strictly as a weak discontinuity using a scalar function:

(10) $$D\left(x\right)=\left\{\begin{array}{c} 1\;\mathrm{at}\;x=x^{*}\\ 0\;\mathrm{at}\;x\neq x^{*} \end{array}\right.$$

where D =1 and D =0 represents interface and matrix or fiber, respectively.

However, the representation of the interface using Equation (10) could always bring challenges to numerical modelling, especially when the topology of the interface become complex. In the interface phase field model, we assume that the transition between the interface and matrix is smooth, and can be expressed using a phase field function η(x):

(11) $$\eta (x)=e^{-\frac{\left|x-x^{*}\right|}{l_{i}}}$$

where $\eta (x^{*})=1$ and η(±∞)=0 represents interface and matrix, respectively. The area with η(x)∈(0,1) represents a transition zone. In Equation (11), the internal length scale $l_{i}$ is used to control the size of the smeared zone. When $l_{i}\to 0$, the smeared representation of Equation (11) degenerates to the discrete representation Equation (10).

The interface phase field function Equation (11) is the optimal solution of the following variational principle:

(12) $$\eta =\mathrm{Arg}\left\{\underset{\eta \in W_{i}}{\inf}\Gamma_{i}\left(\eta \right)\right\}$$

where $W_{i}$ is the boundary condition and $\Gamma_{i}\left(\eta \right)$ is defined by

(13) $$\Gamma_{i}\left(\eta \right)=\frac{1}{2l_{i}}\int \left(\eta^{2}+l_{i}^{2}{\left|\nabla \eta \right|}^{2}\right)dV$$

The variation in Equation (13) with respect to η gives the Euler equation of the interface phase field model and the boundary condition, as specified by

(14) $$\left\{\begin{array}{cc} \eta -l_{i}^{2}\Delta \eta =0, & x\in \Omega^{R}\\ \eta (x)=1, & \;x\in \Gamma_{i}\\ \nabla \eta \cdot n=0, & x\in \partial \Omega^{R} \end{array}\right.$$

where R=1,2,3 represents the space dimension. The fundamental differential equation can be solved with prescribed boundary conditions.

Then, on the basis of the distribution of interface phase field, the equivalent material properties $\Theta_{s}\left(\eta \right)$ in the transition zone can be determined through the following function

(15) $$\Theta_{s}\left(\eta \right)=\{\begin{array}{cc} \;\;\Theta_{i}\left[1-h\left(\eta \right)\right]+\Theta_{m}h\left(\eta \right) & x\geq x^{*}\\ \;\;\Theta_{i}\left[1-h\left(\eta \right)\right]+\Theta_{f}h\left(\eta \right)\; & x<x^{*} \end{array}\;(\Theta =G_{c},\sigma_{\max})$$

where $\Theta_{s}$ represents the equivalent material property in the transition area, and $\Theta_{i}$, $\Theta_{m}$ and $\Theta_{f}$ represent the material parameters of the interface and the other two material components, respectively. $h\left(\eta \right)$ is a transition function, which is defined by

(16) $$h\left(\eta \right)={\left(1-\eta \right)}^{2}$$

it must satisfy:

(17) $$h\left(0\right)=1,\;h\left(1\right)=0,\begin{array}{cc} & h^{\prime }\left(\eta \right) \end{array}<0$$

The last condition of Equation (17) ensures that the material parameters are monotonic after the interface dispersion. The first two conditions guarantee two critical conditions, namely:

(18) $$\Theta_{s}\left(1\right)=\Theta_{i},\;\Theta_{s}\left(0\right)=\left\{\begin{array}{cc} \Theta_{m} & x\geq x^{*}\\ \Theta_{f} & x<x^{*} \end{array}\right.$$

### 2.3. Limitations of Existing Interface Modeling Approaches

**Figure 4:**
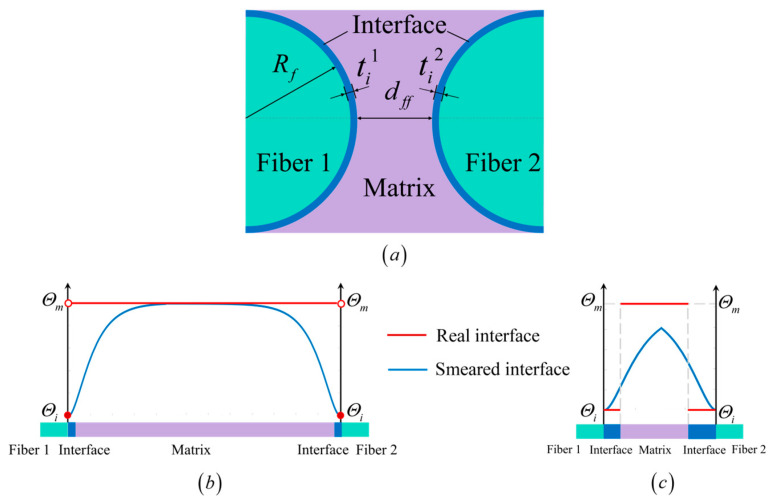
(**a**) Schematic of the composite microstructure. (**b**) Comparison between real and smeared material property distributions when the inter-fiber distance is large and the interface thickness is negligible. (**c**) Comparison between real and smeared material property distributions when the inter-fiber distance is small and the interface thicknesses are different and non-negligible.

To better elucidate the relationship between material property smearing and the microstructure of composite materials, a microstructure is considered, as shown in Figure 4a. Fibers 1 and 2 have the same radius $R_{f}$, with interface thicknesses $t_{i}^{1}$ and $t_{i}^{2}$, respectively. The distance between the two fibers is $d_{ff}$. When $d_{ff}$ is significantly larger than the fiber radius $R_{f}$ and both interface thicknesses are so small that they are difficult to measure, the material property smearing approach described in Section 2.2 yields the distribution shown in Figure 4b. This approach approximates the actual distribution of material properties and alleviates numerical challenges associated with sharp material property discontinuities. Moreover, it only requires the fracture strength and critical energy release rate of the interfacial as input parameters.

In contrast, consider the scenario depicted in Figure 4c, where the interface thicknesses $t_{i}^{1}$ and $t_{i}^{2}$ are different, measurable, and they are non-negligible relative to both the fiber radius $R_{f}$ and the fiber spacing $d_{ff}$. In such cases, the difference in interface thickness is expected to significantly influence structural failure behavior. Applying the conventional smearing method under these conditions, as shown by the blue curve in Figure 4c, leads to a material property distribution that deviates markedly from the actual distribution (red line). Specifically, the material properties within the matrix region are underestimated in terms of fracture strength and critical energy release rate. Furthermore, an artificial peak appears in the smeared property profile between the two fibers. These inaccuracies lead to underestimation of the fracture energy release during failure and overlook essential elastic properties such as the modulus and Poisson’s ratio of the interfacial material, which are not captured by the traditional smearing model.

**Figure 5:**
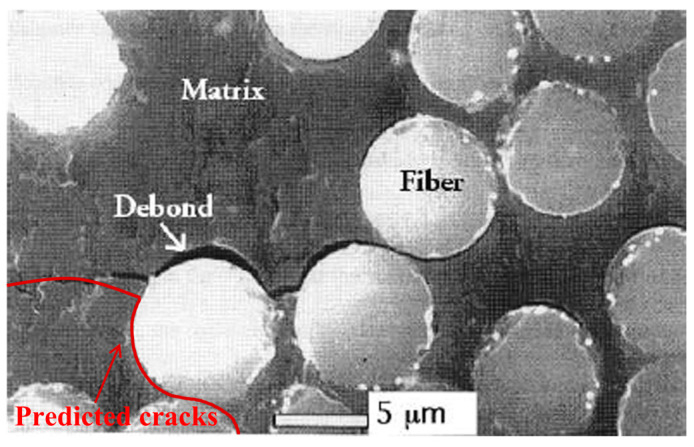
A fracture diagram of an E-glass/carbon/epoxy interlayer hybrid composite [56] (the red line represents the prediction result of the fracture phase field model using the conventional smeared interface method).

However, attempting to address these discrepancies using finer mesh resolution—ensuring the phase field characteristic length $l_{i}$ exceeds five times the minimum mesh size—the number of elements between fibers becomes computationally prohibitive. More critically, the conventional smearing method remains fundamentally incapable of describing the effects of different interface thicknesses. This limitation becomes particularly evident in scenarios such as that shown in Figure 5 [56], where actual crack propagation paths deviate significantly from those predicted by conventional smearing method. Notably, cracks do not necessarily initiate between closely spaced fibers, contradicting the predictions made by the conventional approach. These discrepancies highlight the urgent need for an improved smearing formulation that can simultaneously account for non-uniform interface thicknesses and a complete set of interfacial material properties, thereby enabling more accurate and physically meaningful predictions of failure in composite materials.

## 3. A Novel Unified Framework for Smeared Interface Modeling in Phase-Field Model of Microscopic Fracture

### 3.1. A Novel Interfacial Material Transition Function

Currently, in phase-field modeling of fracture in composite materials with varying interfacial thicknesses, a common approach is to explicitly define the interfacial regions within the structure by directly drawing them based on their actual geometry [43,44,45]. This allows for the investigation of how interface thickness affects the overall structural behavior. While such a method is feasible for structures containing a single fiber or a small number of fibers with regular geometries, it becomes impractical for microstructures containing multiple fibers with irregular shapes and varying interface thicknesses. In such cases, explicitly modeling each interface leads to excessive preprocessing complexity and computational cost. On the other hand, the conventional smearing method has notable limitations—such as its inability to capture spatial variations in interface thickness or to incorporate realistic interfacial material properties—it still offers certain advantages. In particular, it simplifies the modeling process by avoiding the need for explicit interface representation or complex domain partitioning [46,47,48]. This makes it highly effective in scenarios where the interface is extremely thin and its mechanical influence can be neglected. Nevertheless, when the interface thickness becomes non-negligible and varies across fibers, the limitations of the conventional approach become pronounced.

To overcome these limitations, we propose a novel interfacial transition function that retains the advantages of the conventional smearing method while extending its applicability to cases involving different interface thicknesses. This new function enables a more flexible and accurate representation of the interface in complex composite microstructures. The proposed transition function is defined as follows:

(19) $$h_{\mathrm{new}}\left(\eta \right)=\frac{{\left(1-\eta \right)}^{n}}{{\left(1-\eta \right)}^{n}+e^{k(\eta -\eta_{i})}}$$

where n and k are shape-controlling parameters that adjust the profile of the transition function. $\eta_{i}$ is the specified interface phase value, which plays a critical role in controlling the effective thickness of the interface, and is defined as

(20) $$\eta_{i}=e^{-\frac{\left|x_{i}\right|}{l_{i}}}$$

where $x_{i}$ is the specified thickness of the interface in the problem to be calculated and its unit is consistent with that of $l_{i}$.

**Figure 6:**
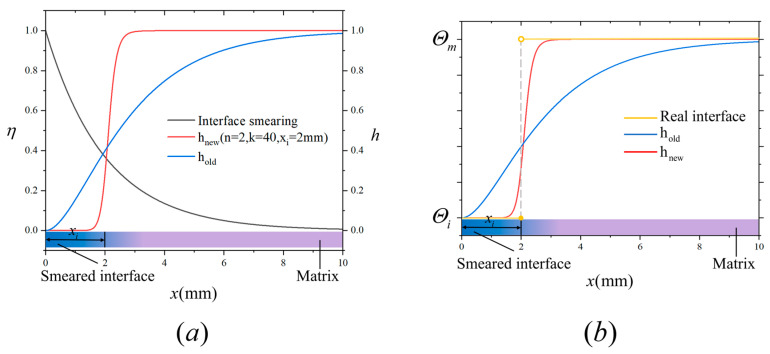
Schematic illustration of interface smearing and material property smearing.(**a**) Interface dispersion results and curves of the two transition functions. (**b**) The distribution of real material properties and the distribution of material properties described by two transition functions.

To evaluate the performance of the proposed interfacial transition function, we consider a one-dimensional bar with a total length of L=20 mm, as illustrated in Figure 3. The interface region is assumed to have a thickness of 4 mm, with the remaining 8 mm on either side representing the fiber and matrix regions, respectively. For analysis, we focus on the right half of the bar, which includes the matrix and half of the interface region, as shown in Figure 6.

A smearing width of $l_{i}=2\;\mathrm{mm}$ is selected, and the resulting distribution of the interfacial phase-field value over the right half of the bar is shown as the black curve in Figure 6a (the left vertical axis). Using this interfacial phase-field value, material properties are smeared across the same region using two different approaches: Equation (16) is shown as the blue curve in Figure 6a (the right vertical axis), representing the conventional transition function (denoted as $h_{\mathrm{old}}$), and Equation (19) is shown as the red curve in Figure 6a (the right vertical axis), representing the proposed transition function (denoted as $h_{\mathrm{new}}$), with parameters n=2, k=40 and $x_{i}=2\;\mathrm{mm}$ (half of the interface thickness). The corresponding material property distributions are shown in Figure 6b as the blue and red curves, respectively. The yellow curve represents the distribution of material properties under the real interface. As observed in Figure 6b, the proposed transition function yields a distribution that not only incorporates interface thickness but also more closely approximates the real variation in material properties. This demonstrates the improved accuracy and flexibility of the proposed approach compared to conventional methods.

**Figure 7:**
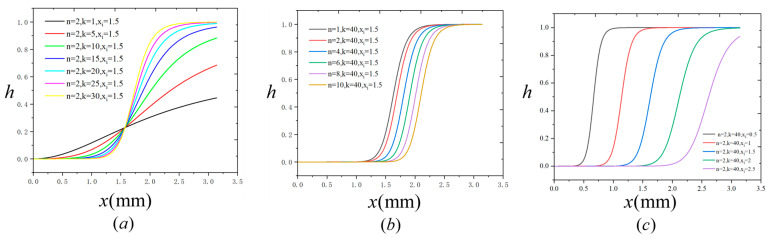
Effects of individual parameters on the shape of the proposed interfacial material transition function: (**a**) Influence of parameter k with fixed n and $x_{i}$; (**b**) Influence of parameter n with fixed k and $x_{i}$; (**c**) Influence of parameter $x_{i}$ with fixed n and k.

To further investigate the influence of individual parameters on the shape of the transition function, we continue analyzing the same problem described above with $l_{i}=2\;\mathrm{mm}$. Using the interfacial phase field distribution obtained earlier, we substitute it into Equation (19) and explore the sensitivity of the function to its three key parameters—n, k and $x_{i}$—by varying one parameter while keeping the other two fixed. The resulting transition function profiles are shown in Figure 7.

In Figure 7a, we fix n=2 and $x_{i}=1.5\;\mathrm{mm}$, and vary k from 1 to 30. This corresponds to an interface thickness of 1.5 mm. As k increases, the transition function becomes increasingly steep, approaching a step-like change, which better approximates the real material transition.

In Figure 7b, we fix k=40 and $x_{i}=1.5\;\mathrm{mm}$, and vary n from 1 to 10. It is observed that increasing n has little effect on the slope of the transition but extends the region where the function value remains near zero. In other words, increasing n effectively shifts the curve to the right without significantly altering its steepness.

In Figure 7c, we fix n=2 and k=40, and vary $x_{i}$ from 0.5 mm to 2.5 mm. This corresponds to studying five different interface thicknesses. As $x_{i}$ increases, the slope of the transition region decreases, resulting in a smoother material transition. This effect becomes particularly pronounced when $x_{i}>l_{i}$, as in the case of $x_{i}=2.5\;\mathrm{mm}$. Based on these observations, it is recommended that the internal length scale of interfacial phase field $l_{i}$ be chosen to be at least twice the interfacial thickness $x_{i}$, to ensure the smeared property distribution accurately reflects the physical material transition.

As discussed in Section 2, when the interface is extremely thin (rendering elastic modulus and Poisson’s ratio of the interfacial material unmeasurable) or when the interface thickness is much smaller than the fiber diameter and the inter-fiber spacing, the conventional method remains a reliable and efficient modeling strategy. To establish consistency between the proposed transition function ($h_{\mathrm{new}}$) and the conventional one ($h_{\mathrm{old}}$) under these assumptions, we perform a parameter fitting procedure involving the three key variables in $h_{\mathrm{new}}$: n, k and $x_{i}$. Specifically, we constrain the fitting to the interval $0<x<5l_{i}$, and enforce that $h_{\mathrm{new}}$ and $h_{\mathrm{old}}$ are exactly equal at the endpoints x=0 and $x=5l_{i}$.

(21) $$h_{\mathrm{old}}\left(0\right)=h_{\mathrm{new}}\left(0;\theta \right)\;h_{\mathrm{old}}\left(5l_{i}\right)=h_{\mathrm{new}}\left(5l_{i};\theta \right)\;\left(\theta =\left[n,k,x_{i}\right]\right)$$

Within this domain, we minimize the mean squared error (MSE) between the two functions, as defined in Equation (22).

(22) $$\underset{\theta }{\min}\;E\left(\theta \right)=\frac{1}{N}\sum_{j=1}^{N}{\left[h_{\mathrm{old}}\left(x_{j}\right)-h_{\mathrm{new}}\left(x_{j};\theta \right)\right]}^{2}\;\left(\theta =\left[n,k,x_{i}\right]\right)$$

**Figure 8:**
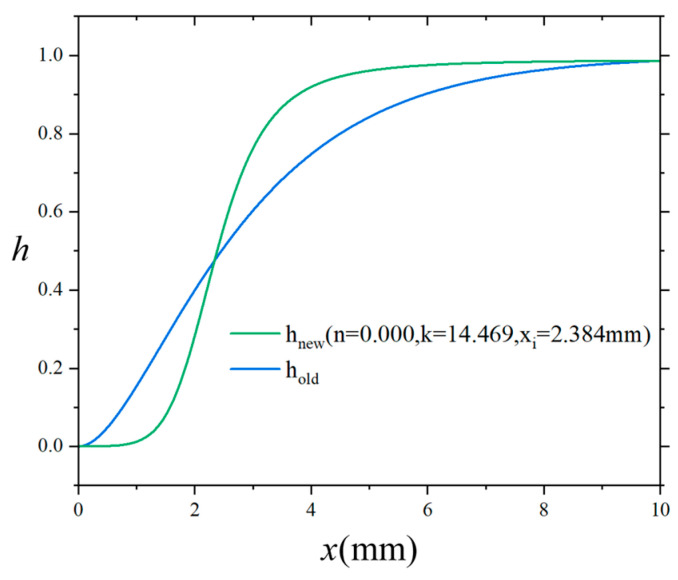
Comparison of the conventional transition function and the proposed method–based fitted transition function.

For example, when $l_{i}=2\;\mathrm{mm}$, the optimal fitting yields n=0.000, k=14.469 and $x_{i}=2.384\;\mathrm{mm}$. The resulting comparison of the two functions is shown in Figure 8. Additionally, Table 2 summarizes the fitted values of n, k and $x_{i}$ for several representative values of $l_{i}$. The effectiveness of this fitted $h_{\mathrm{new}}$ function in replicating the behavior of the original $h_{\mathrm{old}}$ function will be further examined through numerical simulations in Section 4.

Thus, the proposed transition function is capable of addressing two distinct scenarios: Case I, where the fiber–matrix interface is extremely thin and can be considered negligible, and Case II, where varying interface thicknesses significantly affect the structural response. Accordingly, Equation (15) can be reformulated as follows:

(23) $$\Theta_{s}\left(\eta \right)=\left\{\begin{array}{c} \;\;\begin{array}{cc} \Theta_{i}\left[1-h_{\mathrm{new}}\left(\eta \right)\right]+\Theta_{m}h_{\mathrm{new}}\left(\eta \right) & x\geq x^{*} \end{array}\\ \;\;\begin{array}{cc} \Theta_{i}\left[1-h_{\mathrm{new}}\left(\eta \right)\right]+\Theta_{f}h_{\mathrm{new}}\left(\eta \right)\; & x<x^{*} \end{array} \end{array}\right.\left(\Theta =\left\{\begin{array}{cc} G_{c},\sigma_{\max} & \mathrm{Case}\;I\\ E,v,G_{c},\sigma_{\max} & \mathrm{Case}\;\mathrm{II} \end{array}\right.\right)$$

### 3.2. Correction of Interface Energy Release Rate

When the interface debonding process has completed, the energy dissipated per unit area $W_{i}$ must align with the critical energy release rate of the interface $G_{i}$,

(24) $$W_{i}=G_{i}$$

In Figure 3a, the discrete representation of the interface allows for the direct fulfillment of this requirement. When the interface is regularized by a crack phase field as shown in Figure 3b, and assume a crack represented by d takes place at the interface, the dissipated energy can be calculated using the following equation

(25) $$W_{i}=\int_{-\varsigma }^{\varsigma }G_{s}\left(\eta \right)\frac{1}{c_{0}}\left(\frac{\alpha \left(d\right)}{l_{0}}+l_{0}{d^{\prime }}^{2}\right)dV$$

where ς represents the half bandwidth of the crack phase field. The conservation of fracture energy leads to the following relationship

(26) $$G_{i}=\int_{-\varsigma }^{\varsigma }G_{s}\left(\eta \right)\frac{1}{c_{0}}\left(\frac{\alpha \left(d\right)}{l_{0}}+l_{0}{d^{\prime }}^{2}\right)dV$$

Within this equation, the integrand is a result of the multiplication between the equivalent property $G_{s}$ and the surface density functional. The integration of the latter corresponds to the area of the crack surface. Notably, for the current investigation, the equivalent critical energy release rate $G_{s}$ is not a fixed value. instead, it can be determined using Equation (23) as presented by

(27) $$G_{s}\left(\eta \right)=\left\{\begin{array}{c} \;\;\begin{array}{cc} G_{i}\left[1-h\left(\eta \right)\right]+G_{m}h\left(\eta \right)\; & x\geq x^{*} \end{array}\\ \;\;\begin{array}{cc} G_{i}\left[1-h\left(\eta \right)\right]+G_{i}h\left(\eta \right)\;\;\; & x<x^{*} \end{array} \end{array}\right.$$

When $G_{m}>G_{i}$ and $G_{f}>G_{i}$, we have $G_{s}>G_{i}$, thereby

(28) $$\int_{-\varsigma }^{\varsigma }G_{s}\left(\eta \right)\frac{1}{c_{0}}\left(\frac{\alpha \left(d\right)}{l_{0}}+l_{0}{d^{\prime }}^{2}\right)dV>\int_{-\varsigma }^{\varsigma }G_{i}\left(\eta \right)\frac{1}{c_{0}}\left(\frac{\alpha \left(d\right)}{l_{0}}+l_{0}{d^{\prime }}^{2}\right)dV=G_{i}$$

It is evident from the above inequality that incorporating Equation (15) directly into the interface dispersion equation fails to uphold the equivalence condition of fracture energy. Similarly, the equivalent condition may not be satisfied when $G_{m}\neq G_{i}$ and $G_{f}\neq G_{i}$. Therefore, a generalized interface critical energy release rate ${\bar{G}}_{i}$ is introduced here to replace the real energy release rate on the interface, forcing the value of Equation (24) to meet the interface fracture energy equivalence.

(29) $$\begin{array}{ll} G_{i}= & \int_{-\varsigma }^{x^{*}}\left\{{\bar{G}}_{i}\left[1-h\left(\eta \right)\right]+G_{m}h\left(\eta \right)\right\}\frac{1}{c_{0}}\left(\frac{\alpha \left(d\right)}{l_{0}}+l_{0}{d^{\prime }}^{2}\right)dV\\ & +\int_{x^{*}}^{\varsigma }\left\{{\bar{G}}_{i}\left[1-h\left(\eta \right)\right]+G_{f}h\left(\eta \right)\right\}\frac{1}{c_{0}}\left(\frac{\alpha \left(d\right)}{l_{0}}+l_{0}{d^{\prime }}^{2}\right)dV \end{array}$$

The generalized interface critical energy release rate ${\bar{G}}_{i}$ is obtained [46,48,57],

(30) $${\bar{G}}_{i}=\frac{G_{i}-G_{m}\int_{-\varsigma }^{x^{*}}h\left(\eta \right)\;\frac{1}{c_{0}}\left(\frac{\alpha \left(d\right)}{l_{0}}+l_{0}{d^{\prime }}^{2}\right)dV-G_{f}\int_{x^{*}}^{\varsigma }h\left(\eta \right)\;\frac{1}{c_{0}}\left(\frac{\alpha \left(d\right)}{l_{0}}+l_{0}{d^{\prime }}^{2}\right)dV}{\int_{-\varsigma }^{\varsigma }\left[1-h\left(\eta \right)\right]\frac{1}{c_{0}}\left(\frac{\alpha \left(d\right)}{l_{0}}+l_{0}{d^{\prime }}^{2}\right)\;dV}$$

This equation can be solved numerically in practice.

### 3.3. Finite Element Formulation

The finite element method is employed to discretize the interface phase field, the fracture phase field and the displacement field, as expressed in Equation (31).

(31) $$\eta =N^{\eta }\eta^{e}\;\;\;\;\;\;\;\;\;d=N^{d}d^{e}\;\;\;\;\;\;\;\;\;u\;=\;N^{u}u^{e}$$

where $N^{\eta }$, $N^{d}$ and $N^{u}$ represent standard shape function matrices for the interface phase field, the fracture phase field and the displacement field, respectively. The vectors $\eta^{e}$, $d^{e}$ and $u^{e}$ correspond to the nodal interface phase field, the nodal fracture phase field and the nodal displacement of an element. The gradient of the interface phase field ∇η, the gradient of the fracture phase field ∇d, the strain vector ε are defined as follows:

(32) $$\nabla \eta =B^{\eta }\eta^{e}\;\;\;\;\;\;\nabla d=B^{d}d^{e}\;\;\;\;\;\;\varepsilon \;=\;B^{u}u^{e}$$

where the matrix $B^{\eta }$, $B^{d}$ and $B^{u}$ are the derivatives of the shape function matrices. Their forms can be obtained through the conventional manner, thus not listed here for simplicity. The corresponding residual vector of the interface phase field, the fracture phase field and the displacement field can be expressed by:

(33) $$R^{\eta }=\sum_{e}\left(\int_{\Omega^{e}}\frac{\eta^{e}}{l_{i}^{2}}{\left[N^{\eta }\right]}^{T}dV+\int_{\Omega^{e}}\left[{\left[B^{\eta }\right]}^{T}B^{\eta }\eta^{e}\right]dV\right)$$

(34) $$R^{d}=\sum_{e}\left(\int_{\Omega^{e}}\omega^{\prime }\left(d\right)H{\left(N^{d}\right)}^{T}dV+\int_{\Omega^{e}}\frac{G_{c}}{c_{0}}\left[\frac{\alpha^{\prime }\left(d\right)}{l_{0}}{\left(N^{d}\right)}^{T}+2l_{0}{\left(B^{d}\right)}^{T}B^{d}d^{e}\right]dV\right)$$

(35) $$R^{u}=\sum_{e}\left(\int_{\Omega^{e}}{\left[N^{u}\right]}^{T}fdV+\int_{\partial \Omega_{t}^{e}}{\left[N^{u}\right]}^{T}tdS-\int_{\Omega^{e}}\omega \left(d\right)\left[{\left(B^{u}\right)}^{T}DB^{u}u^{e}\right]dV\right)$$

where $\underset{e}{\Sigma }$ represents element assembly, D are the stiffness matrix. The tangential stiffness matrices at the lth increment step can be expressed by

(36) $$K_{l}^{\eta }=\sum_{e}\left(\int_{\Omega^{e}}\frac{1}{l_{i}^{2}}{\left[N^{\eta }\right]}^{T}dV+\int_{\Omega^{e}}{\left[B^{\eta }\right]}^{T}B^{\eta }dV\right)$$

(37) $$K_{l}^{dd}=\sum_{e}\left(\int_{\Omega^{e}}\omega^{\prime \prime }\left(d\right)H{\left(N^{d}\right)}^{T}N^{d}dV+\int_{\Omega^{e}}\frac{G_{c}}{c_{0}}\left[\frac{\alpha^{\prime \prime }\left(d\right)}{l_{0}}{\left(N^{d}\right)}^{T}N^{d}+2l_{0}{\left(B^{d}\right)}^{T}B^{d}\right]dV\right)$$

(38) $$K_{l}^{uu}=\sum_{e}\left(\int_{\Omega^{e}}\omega \left(d\right){\left[B^{u}\right]}^{T}{\left(D\right)}_{l}^{e}B^{u}dV\right)$$

(39) $$K_{l}^{ud}=K_{l}^{du}=0$$

where ${\left(D\right)}_{l}^{e}$ is the stiffness matrix of the element at the lth increment step describing the relationship between stress and total strain. In a certain incremental step, the iteration scheme of the interface phase filed can be written as

(40) $${\left\{\eta \right\}}_{l+1}={\left\{\eta \right\}}_{l}-{\left[K_{l}^{\eta }\right]}^{-1}{\left\{R^{\eta }\right\}}_{l}$$

The iteration scheme of the fracture phase filed and the displacement field coupling can be written as

(41) $${\left\{\begin{array}{c} u\\ d \end{array}\right\}}_{l+1}={\left\{\begin{array}{c} u\\ d \end{array}\right\}}_{l}-{\left[\begin{array}{cc} K_{l}^{uu} & \\ & K_{l}^{dd} \end{array}\right]}^{-1}{\left\{\begin{array}{c} R^{u}\\ R^{d} \end{array}\right\}}_{l}$$

In the present method, we have an equivalent staggered iteration form. And we assume a hybrid formulation proposed by Ambati to calculate and degrade the displacement field, which not only guarantees the same result as the original formulation, but also delivers a saving in computational cost of about one order of magnitude [40].

## 4. Verification

**Figure 9:**
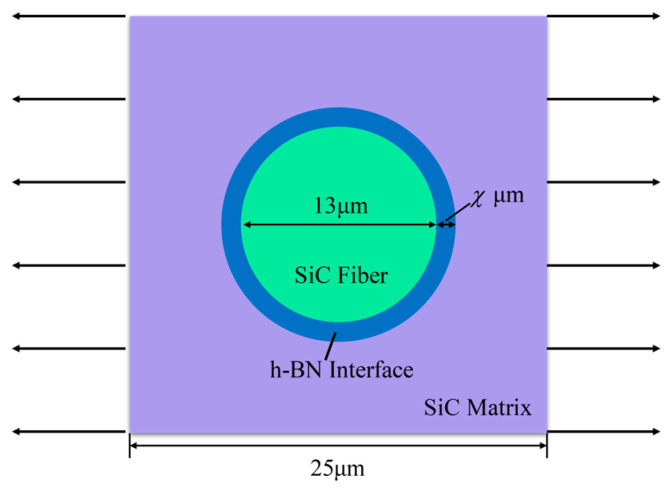
Single-fiber representative volume element model and loading condition.

To validate the proposed unified framework for smeared interface modeling, this section considers a representative volume element (RVE) of a single-fiber SiC_f_/SiC_m_ composite with an h-BN interface subjected to transverse tensile loading, as illustrated in Figure 9. The typical material properties of the SiC_f_/SiC_m_ composite constituents, including the h-BN interface, are listed in Table 3 [43,58]. Following the assumptions made in [43], all materials are treated as isotropic for modeling purposes. Although h-BN is intrinsically an anisotropic layered material, exhibiting distinct mechanical properties in the transverse and longitudinal directions (as reported in Table 3), it is modeled here as isotropic using its transverse mechanical properties. This simplification is justified by the relatively thin interface and the application of transverse tensile loading in this case. The RVE model has dimensions of 25 μm×25 μm, with a fiber diameter of 13 μm, consistent with commonly reported values. The thickness of the h-BN interface is varied systematically across five cases: 0.1 μm, 0.25 μm, 0.5 μm, 0.75 μm, and 1 μm. These configurations not only allow for quantitative evaluation of the proposed method’s accuracy in capturing the mechanical response across different interface thicknesses, but also it easier to understand how interface thickness affects the transverse tensile strength of the composite. Understanding this relationship is essential in the design and optimization of SiC_f_/SiC_m_ composites, where the interface plays a critical role in damage initiation, crack deflection, and overall structural performance.

**Figure 10:**
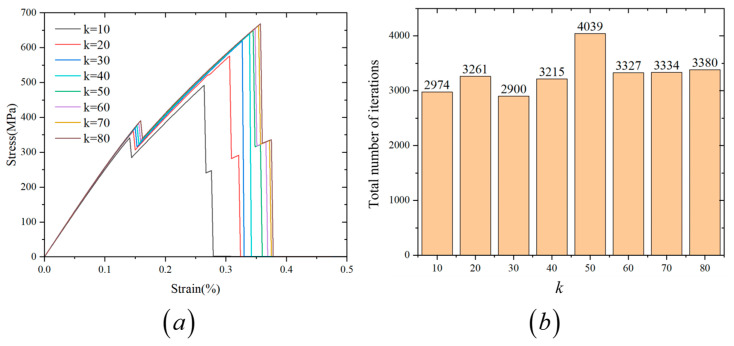
(**a**) Stress–strain curves under different values of k with fixed n and $x_{i}$. (**b**) Total number of iterations required for different values of k with fixed n and $x_{i}$.

Before performing the simulations, it is necessary to determine appropriate values for the parameters of the proposed transition function. As established in Section 3.1, the shape of the proposed transition function is governed by three parameters: n, k and $x_{i}$. Among these, $x_{i}$ corresponds directly to the physical thickness of the interface in the composite. To ensure numerical stability, $x_{i}$ must remain smaller than the internal length scale of interfacial phase field $l_{i}$. Based on the parametric study shown in Figure 7b, the function is relatively insensitive to changes in n, and a value of n=2 is found to provide sufficient smoothness and accuracy. However, the parameter k strongly influences the steepness of the transition function and, therefore, requires further investigation. To determine an appropriate value for k, we consider a test case with an interface thickness of $x_{i}=0.5\;\mu m$, while keeping n=2 fixed. Use the internal length scale of interfacial phase field $l_{i}=2\;\mu m$ to smear the interface. A series of simulations is conducted with k values ranging from 10 to 80 (specifically, k=10,20,30,40,50,60,70,80). The resulting stress–strain curves are shown in Figure 10a. It is important to note that changes in k alter the steepness of the material transition, which in turn affects the convergence behavior during computation. To assess this, we record the total number of nonlinear iterations required under a fixed number of loading steps for each value of k. These results are plotted in Figure 10b. From Figure 10a, it is observed that the simulation results tend to converge as k increases. When k≥60, the stress–strain response becomes nearly unchanged, indicating numerical convergence. Meanwhile, Figure 10b shows that the total number of iterations stabilizes for k≥60. Therefore, to balance accuracy and computational efficiency, a value of k=70 is selected for all subsequent simulations in this study. In summary, the transition function parameters used throughout this work are fixed as n=2 and k=70, unless otherwise specified.

**Figure 11:**
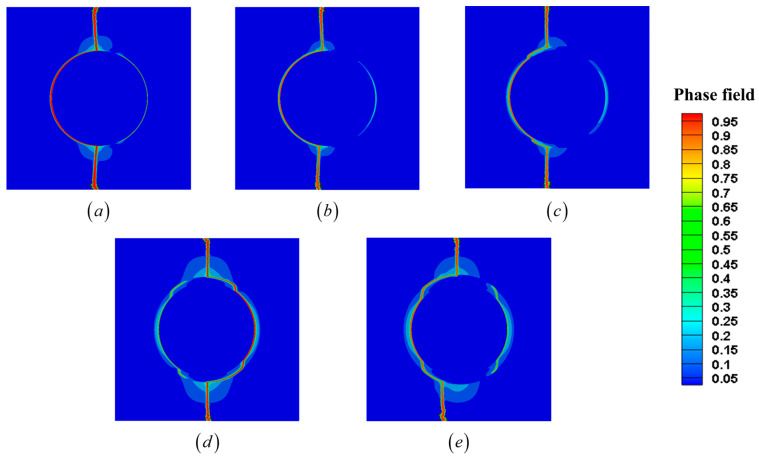
Predicted crack patterns under different interface thicknesses. (**a**) 0.1 μm. (**b**) 0.25 μm. (**c**) 0.5 μm. (**d**) 0.75 μm. (**e**) 1 μm.

**Figure 12:**
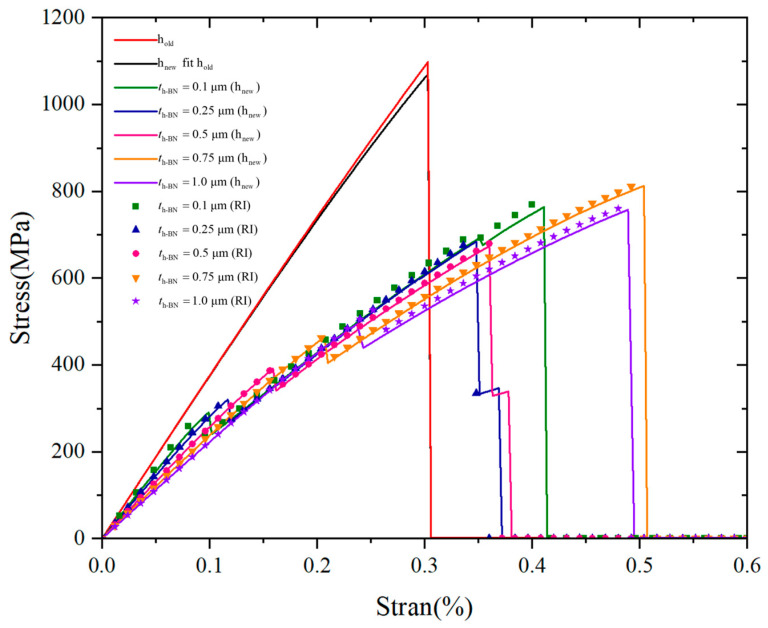
Stress–strain response of SiC_f_/SiC_m_ composites under transverse tensile for the different interface thickness.

A series of simulations was performed to evaluate the performance and flexibility of the proposed interfacial transition function ($h_{\mathrm{new}}$) under varying interface thicknesses. The internal length scale of interfacial phase field was fixed at $l_{i}=2\;\mu m$, and the internal length scale of fracture phase field was set to $l_{0}=0.06\;\mu m$. For each case using $h_{\mathrm{new}}$, the parameter $x_{i}$ was set to 0.1 μm, 0.25 μm, 0.5 μm, 0.75 μm, and 1 μm. To provide a reliable reference for accuracy assessment, a set of models was constructed in which the interface regions were explicitly defined in the geometry to reflect their real thickness. These models, referred to as RI (real interface), serve as the benchmark solution, as they directly represent the physical geometry and material interfaces of the system. For comparison, a second set of simulations was carried out using the traditional smearing approach ($h_{\mathrm{old}}$) described in Section 2.2. $h_{\mathrm{old}}$ cannot capture the influence of varying interface thickness, and therefore produces only a single result. In addition, an auxiliary case was included where the parameters of $h_{\mathrm{new}}$ were fitted to reproduce the result of $h_{\mathrm{old}}$ under thin-interface conditions (specifically, at $l_{i}=2\;\mu m$ described in Section 3.1). This case, denoted as $h_{\mathrm{new}}$ fit $h_{\mathrm{old}}$. The predicted crack patterns obtained using the proposed interfacial transition function ($h_{\mathrm{new}}$) are shown in Figure 11. All simulations in this section are conducted using the AT2 phase field model, which is widely adopted in the literature as a baseline fracture formulation. The quadratic degradation function ensures smooth crack evolution and stable numerical implementation, making it a suitable choice for verification studies and for capturing general brittle fracture behavior. The elastic potential is decomposed into tensile and compressive components following the formulation given in Equation (5). The corresponding stress–strain curves for all simulated cases are presented in Figure 12.

The predicted stress–strain responses using $h_{\mathrm{new}}$ are compared with those obtained from the real interface (RI) models, which serve as the benchmark for accuracy. As shown in Figure 12, the stress–strain curves generated by $h_{\mathrm{new}}$ closely follow those of RI across all interface thicknesses. Although the peak strength in $h_{\mathrm{new}}$ predictions is consistently slightly lower than that of RI, the overall curve shapes—such as stiffness, peak location, and post-peak behavior—show a high degree of consistency. This systematic underestimation is expected and can be attributed to the smoothing effect inherent in the smeared transition formulation, which effectively reduces local stress concentrations and leads to a slightly softened mechanical response. Nevertheless, the good agreement confirms that $h_{\mathrm{new}}$ provides an accurate and reliable approximation of the mechanical behavior produced by more detailed geometric interface models. In terms of computational efficiency, the advantage of $h_{\mathrm{new}}$ is significant. The RI approach, while accurate, requires a separate geometry and mesh for every interface thickness, which becomes impractical when calculating the microstructure of composite materials with a large number of fibers and interfaces. In contrast, $h_{\mathrm{new}}$ achieves this flexibility within a single model and mesh by simply modifying the parameters n, k and $x_{i}$. This greatly simplifies the simulation process, reduces pre-processing time, and makes parametric studies much more accessible.

When compared to the conventional smearing approach ($h_{\mathrm{old}}$), the benefits of $h_{\mathrm{new}}$ become even more apparent. As shown in Figure 12, the $h_{\mathrm{old}}$ curve significantly deviates from the benchmark in both slope and peak stress. Since $h_{\mathrm{old}}$ assumes a zero-thickness interface, it cannot capture the transition in material properties, nor can it reflect the effects of finite interface geometry. This leads to poor approximation of both stiffness and strength. Furthermore, it offers no capacity for adjusting interface thickness, making it unsuitable for studies focused on interface design. The resulting curve of $h_{\mathrm{new}}$ fit $h_{\mathrm{old}}$ is almost identical to the original $h_{\mathrm{old}}$ result, verifying that $h_{\mathrm{new}}$ retains compatibility with $h_{\mathrm{old}}$ in the appropriate limit. This reinforces the robustness and versatility of $h_{\mathrm{new}}$, as it is capable of covering both thin- and finite-thickness interface scenarios within a unified framework.

Beyond numerical accuracy, the simulations also reveal valuable insights into how interface thickness influences crack propagation and transverse strength. As shown in Figure 12, the maximum transverse tensile strength is observed when the interface thickness is 0.75 μm. The corresponding crack patterns in Figure 11 show that, at this thickness, the crack deflects in the interface region before reaching the matrix region. This crack deflection increases the energy dissipation during fracture, delays complete failure, and results in higher load-bearing capacity. This behavior is characteristic of toughening mechanisms in ceramic matrix composites, where the presence of a soft interface allows cracks to deviate and spread rather than propagate straight to the matrix. When the interface is thick enough to enable significant stress redistribution but not so thick as to weaken the material excessively, it contributes positively to fracture resistance. In contrast, for thinner interfaces (<0.5 μm), the crack path shows little to no deflection, often propagating directly to the matrix. Although these cases still include interfacial material, the thickness is insufficient to trigger meaningful crack deviation. At the same time, the addition of a soft interfacial material reduces the overall stiffness and strength slightly compared to the zero-thickness case, which explains why the transverse strength is lower for very thin interfaces. Interestingly, although increasing the interface thickness beyond 0.75 μm continues to support crack deflection, it eventually begins to reduce overall strength. At 1 μm, the transverse strength declines slightly compared to 0.75 μm, though it remains higher than the strength in the 0.1 μm case. This suggests a trade-off: as the interface becomes too thick, its inherent softness begins to dominate, weakening the structural integrity more than it enhances toughness. Taken together, these results suggest that an optimal interface thickness exists, balancing two competing effects: (i) crack deflection and energy dissipation, which tend to increase strength, and (ii) stiffness reduction due to excessive interfacial softness, which tends to lower it. Based on our simulations, this optimal thickness falls into the range of $\left[0.75\;\mu m\;,\;1\;\mu m\right)$. Further refinement within this range may help identify the precise interface design that maximizes transverse strength and fracture resistance in SiC_f_/SiC_m_ composites.

## 5. Validation

**Figure 13:**
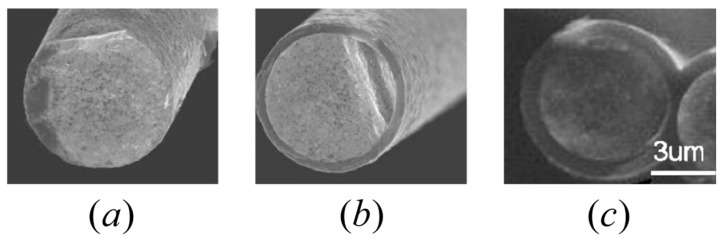
Cross-sections of uncoated and PyC coated SA fibers formed with CVD technique: (**a**) uncoated, (**b**) CVD-0.50 and (**c**) CVD-1.00 [59].

SiC_f_/SiC_m_ composites offer excellent high-temperature performance and low neutron activation, making them ideal for advanced nuclear systems. However, their fracture toughness remains highly sensitive to the fiber–matrix interface properties. To address this and to optimize the balance between high flexural strength and pseudo-ductile fracture behavior, Shimoda et al. [59,60] investigated the influence of PyC interface thickness on the microstructure and mechanical properties of unidirectional SiC_f_/SiC_m_ composites fabricated using the Nano-Infiltration and Transient Eutectic-phase (NITE) process. By precisely controlling the thickness of PyC layer via chemical vapor deposition (CVD) process, they produced composites with interfaces thicknesses of 0 μm (Uncoated), 0.5 μm (CVD-0.50), and 1 μm (CVD-1.00), and systematically evaluated the resulting density, porosity, crack paths, and tensile strength. Figure 13 shows field emission scanning electron microscopy (FE-SEM) images of uncoated and PyC coated fibers formed with CVD coating technique. Most filaments in the tows through CVD were well coated, with the thickness of appropriately 0.5 μm and 1 μm, which respectively called CVD-0.50 and CVD-1.00. In the experimental study [59], the SiC_f_/SiC_m_ composites were fabricated using unidirectional Tyranno^TM^-SA fibers as reinforcement, which have a diameter of approximately 7.5 μm. The matrix was formed by nano-sized SiC powder densified through NITE process. The PyC interface between fiber and matrix was deposited by CVD process, with thicknesses precisely controlled at 0 μm, 0.5 μm and 1 μm. The corresponding volume fractions of fibers and pores under each interface condition are listed in Table 4.

**Figure 14:**
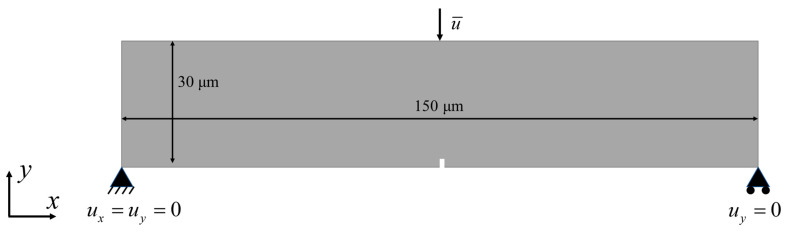
The geometry and boundary conditions of three-point bending test.

**Figure 15:**
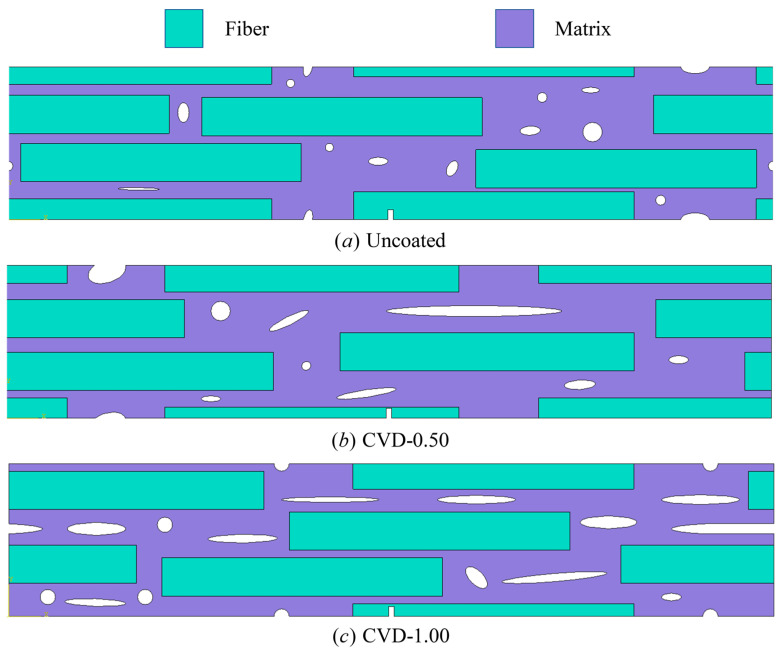
Two-dimensional RVE models of SiC_f_/SiC_m_ composites under different PyC interface thickness conditions. (**a**) composites with interfaces thicknesses of 0 μm (Uncoated). (**b**) composites with interfaces thicknesses of 0.5 μm (CVD-0.50). (**c**) composites with interfaces thicknesses of 1 μm (CVD-1.00).

**Figure 16:**
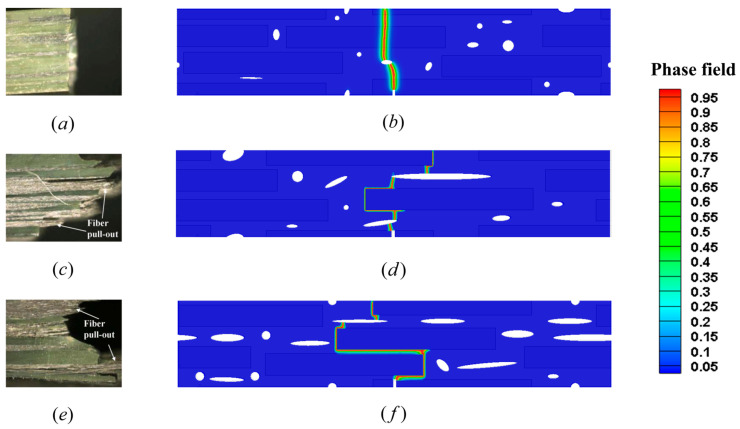
Comparison between experimental fracture images and simulation results of SiC_f_/SiC_m_ composites with different PyC interface thicknesses under three-point bending: (**a**) Experimental result for uncoated case. (**b**) Simulation result for uncoated case. (**c**) Experimental result for CVD-0.50 case. (**d**) Simulation result for CVD-0.50 case. (**e**) Experimental result for CVD-1.00 case. (**f**) Simulation result for CVD-1.00 case.

**Figure 17:**
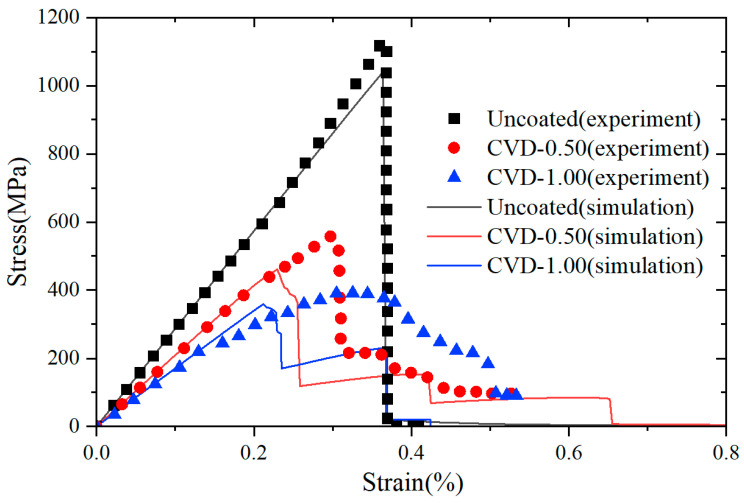
Flexural stress–strain curves of SiC_f_/SiC_m_ composites with various PyC interface thickness.

Inspired by their findings, we performed a set of phase field simulations to replicate these interface configurations and validate the proposed smeared interface transition model ($h_{\mathrm{new}}$). In [59], the three-point bending tests were performed using an INSTRON 5581 universal testing machine with rectangular bar specimens of dimensions 4.0 mm (width) × 25.0 mm(length) × 2.0 mm(thickness). A crosshead speed of 0.5 mm/min and an outer support span of 18.0 mm were applied, in accordance with standard three-point bending practice. Between three and five specimens were tested for each condition to ensure statistical reliability. The same mechanical loading configuration was adopted to evaluate the flexural strength. The specimen geometry was modeled with dimensions of 150 μm×30 μm, as shown in Figure 14. Boundary and loading conditions followed the standard three-point bending layout. The stress–strain response was calculated using the conventional bending formula:

(42) $$\begin{array}{l} \sigma =\frac{3Pl}{2wt^{2}}\\ \varepsilon =\frac{6Dt}{l^{2}} \end{array}$$

where P is load at a point of deflection of a load–displacement curve in test, l is outer support span, w is specimen width, t is specimen thickness, D is deflection at beam center at a given point in the test. The microstructural models used in these simulations are shown in Figure 15. Based on the constituent ratios in Table 4, a RVE was constructed in which fibers, pores, and matrix are randomly distributed. Periodic microstructural features were imposed such that the fiber and pore morphology on opposing edges (left–right and top–bottom) align continuously, satisfying RVE conditions. The fiber diameter and volume fraction were matched to the experimental data. The internal length scale of interfacial phase field was fixed at $l_{i}=2\;\mu m$. The internal length scale of fracture phase field was set to $l_{0}=0.6\;\mu m$ in Uncoated case and CVD-1.00 case, and the internal length scale of fracture phase field was set to $l_{0}=0.2\;\mu m$ in CVD-0.50 case. The material properties of the SiC_f_/SiC_m_ composite constituents, including the PyC interface, are listed in Table 5. The transition function parameters used for the CVD-0.50 case are n=2, k=70 and $x_{i}=0.5\;\mu m$, while for the CVD-1.00 case, the parameters are n=2, k=70 and $x_{i}=1\;\mu m$, respectively. All simulations in this section are conducted using the PF-CZM model with linear degradation. This model is more convenient for analyzing interface-driven damage in composites because it provides a closer link to cohesive-zone traction–separation laws. The linear degradation directly connects fracture energy dissipation with interfacial strength parameters, thereby enabling a more accurate representation of interfacial fracture phenomena. The elastic potential is decomposed into tensile and compressive components following the formulation given in Equation (6), The simulated fracture patterns and the corresponding experimental fracture images are shown in Figure 16, and the comparison of stress–strain curves obtained from experiments and numerical simulations for all cases is presented in Figure 17.

As shown in Figure 16, both the experimental and simulated fracture surfaces for the Uncoated case exhibit relatively smooth fracture surfaces. In this case, damage occurred in both the fibers and the matrix. No fiber pull-out or crack deflection is observed, indicating that the absence of a soft interface causes the crack to propagate directly across the composite cross-section. This behavior is reflected in the stress–strain curve (Figure 17, black square point set and black line), which shows a steep linear increase followed by abrupt failure, characteristic of brittle fracture. The corresponding simulation reproduces this behavior well, capturing both the fracture mode and the overall stiffness response. This sudden brittle failure, typical of uncoated composites, poses a serious limitation in structural applications, as it lacks any forewarning before final fracture, making failure unpredictable and potentially unsafe under service conditions.

In contrast, the CVD-0.50 case reveals distinct signs of crack deflection and fiber pull-out in both experimental and numerical results. The simulation accurately predicts that the crack path is redirected along the interface rather than penetrating the fibers directly. This redirection is enabled by the presence of a relatively weak PyC interface, which accommodates debonding under loading. The stress–strain curve (Figure 17, red dots point set and red line) exhibits a more gradual post-peak softening, indicating pseudo-ductile behavior, in close agreement with experimental observations [59]. Notably, the maximum stress is lower than that of the Uncoated case. Fractographic evidence confirms that this configuration leads to the most favorable combination of toughness and strength, as it activates multiple toughening mechanisms such as crack deflection, fiber bridging, and interfacial sliding [65,66].

For the CVD-1.0 case, the simulated crack morphology remains similar to that of the CVD-0.50 case, with significant crack deflection and interface failure. However, the increased thickness of the interface leads to a reduction in matrix continuity and potentially increases the risk of interfacial degradation under thermal or chemical exposure. Experimentally, this case shows a slightly lower flexural strength than the CVD-0.50 composite (Figure 17, red dots and blue triangles point set), and the simulation similarly predicts a modest decrease in peak load. The stress–strain response (Figure 17, blue line) shows a pseudo-ductile trend, but with reduced stiffness and peak strength compared to the CVD-0.50 case.

The simulation results are in strong agreement with experimental observations, reaffirming the critical role of interfacial design in SiC_f_/SiC_m_ composites. Specifically, the model successfully reproduces the key finding reported in the referenced work [59]: A better balance between high flexural strength and pseudo-ductile fracture behavior is achieved when a PyC interface thickness of approximately 0.5 μm is used, enabling effective crack deflection and fiber pull-out. These results confirm that the thickness and condition of the PyC interface significantly influence microstructural evolution and dominate the mechanical and fracture responses of the composite system.

From Figure 17, it can be observed that the experimental and numerical results show good agreement in the Uncoated case. In contrast, for the CVD-0.50 and CVD-1.00 cases, the experimental results exhibit significantly higher strengths compared to the numerical predictions. Quantitative comparisons of the flexural strengths are summarized in Table 6, along with their corresponding relative errors. The Uncoated case exhibits excellent agreement between simulation and experiment, with relative error below 10%, confirming the validity of the model in predicting brittle fracture behavior. However, for the CVD-0.50 and CVD-1.00 cases, the relative errors exceed 10%. This discrepancy is primarily attributed to differences between the nominal and real interface thicknesses in the experimental composites. As reported by Shimoda et al. [59], the PyC layers deposited during the CVD process underwent partial degradation during hot pressing, due to chemical reactions with oxide sintering additives, as described by the following thermodynamically favorable reaction mechanism [67,68]:

(43) $$3{C(s)+\mathrm{SiO}}_{2}(s,l)=\mathrm{SiC}(s)+2\mathrm{CO}(g)$$

As a result, the real interfaces in the experiments were thinner than modeled, thereby yielding higher strength than predicted numerically.

Despite this, the model successfully captures the dominant fracture mechanisms in all three cases. It reproduces key features such as brittle fracture in the uncoated condition, pseudo-ductile response with deflection in the CVD-0.50 case and CVD-1.00 case, demonstrating the versatility and robustness of the proposed smeared-interface approach. Furthermore, the numerical method offers significant advantages over purely experimental approaches. For instance, while the experimental setup requires fabricating separate specimens with different interface geometries, the numerical model achieves this by adjusting only the transition function parameters in a single RVE mesh. This reduces modeling workload, eliminates geometric meshing challenges near thin interfaces, and allows efficient exploration of a broader parameter space—including interface thicknesses that may be difficult or impossible to fabricate precisely in practice.

In conclusion, the proposed phase-field framework, incorporating a flexible interfacial transition function, proves capable of capturing the microstructural fracture responses of SiC_f_/SiC_m_ composites across a range of interface thicknesses. It offers both accuracy and computational efficiency, making it a powerful tool for guiding interface design in advanced structural ceramics.

## 6. Conclusions

This work proposes a novel unified framework for smeared interface modeling in phase-field simulations of microscopic fracture, capable of accurately capturing the influence of variable interface thickness across composite microstructures. A new transition function governed by three parameters n, k and $x_{i}$ is developed to describe the spatial variation in material properties within the interface zone. The role of each parameter in shaping the material transition was systematically analyzed, and a simplified single fiber example was used to illustrate a practical guideline for parameter selection. In this study, the parameters were determined as n=2, k=70, while $x_{i}$ was directly set according to the actual interface thickness.

The proposed model was then applied to simulate transverse tensile failure in a ceramic matrix composite with an h-BN interface. Five cases with different interface thicknesses (0.1 μm, 0.25 μm, 0.5 μm, 0.75 μm, and 1 μm) were studied to determine the optimal interface thicknesses for maximizing transverse strength. Results revealed that the highest strength was achieved when the interface thickness falls into the range of $\left[0.75\;\mu m\;,\;1\;\mu m\right)$. A benchmark comparison with real interface models confirmed the accuracy and reliability of the proposed smeared interface approach. Furthermore, by fitting the conventional smearing function with the proposed one, it was demonstrated that the new method retains the capability of classical models while extending their applicability to more complex scenarios.

In the validation stage, the model was employed to replicate the experiments reported in [59], where the influence of PyC interface thickness on the flexural strength and fracture behavior of ceramic composites was studied. The numerical simulations not only reproduced the key experimental trends, including pseudo-ductile fracture at 0.5 μm PyC thickness, but also confirmed that this thickness offers an optimal balance between interfacial protection and crack deflection.

Finally, to further demonstrate the generality of the proposed framework, the smeared-interface method was applied within two different phase-field formulations (AT2 and PF-CZM with linear degradation). The consistent performance across these cases shows that the method is not restricted to a specific phase-field model but can be extended broadly to different formulations for composite fracture simulations.

In conclusion, the proposed method provides a more comprehensive and physically meaningful framework for modeling fiber–matrix interfaces in composite microstructures, enhancing both the predictive capability and practical flexibility of phase-field simulations across a wide range of composite material systems.

## Figures and Tables

**Table 1:** The specific functional expressions and parameters of the two phase-field models.

Model	AT2	PF-CZM			
α(d)	$d^{2}$	$2d-d^{2}$			
$c_{0}$	2	π			
ω(d)	${\left(1-d\right)}^{2}$	$\frac{{\left(1-d\right)}^{p}}{{\left(1-d\right)}^{p}+Q(d)}$ $\left(Q(d)=a_{1}d(1+a_{2}d+a_{2}a_{3}d^{2})\right)$	Type	Linear	Cornelissen
			p	2	2
			$a_{1}$	$\frac{4EG_{c}}{\pi l_{0}\sigma_{\max}^{2}}$	$\frac{4EG_{c}}{\pi l_{0}\sigma_{\max}^{2}}$
			$a_{2}$	−0.5	1.3868
			$a_{3}$	0	0.6567

Note: E is Young’s modulus, $\sigma_{\max}$ is the material strength.

**Table 2:** Fitting parameters n, k and $x_{i}$ for $h_{\mathrm{new}}$ at different $l_{i}$.

$l_{i}$	n	k	$x_{i}$
0.1	0.000	14.469	0.596
0.5	0.000	14.469	1.192
1.0	0.000	14.469	1.788
1.5	0.000	14.469	2.384
2.0	0.000	14.469	2.979

**Table 3:** Properties of HiPerComp SiC fiber, SiC matrix, and h-BN interface [43,58].

Material Property	SiC Fiber	SiC Matrix	h-BN Interface
Young’s modulus E	380 GPa	360 GPa	10/360–800 GPa
Poisson’s ratio υ	0.185	0.185	0.005/0.18
Critical energy release rate $G_{c}$	$17{\;J/m}^{2}$	${8\;J/m}^{2}$	$1.7/5–131{\;J/m}^{2}$
Fracture strength $\sigma_{\max}$	2600 MPa	800 MPa	75/800 MPa

**Table 4:** Fiber volume, porosity and density of hot-pressed SiC_f_/SiC_m_ composites with various interface conditions [59].

	Fiber Volume (%)	Open Porosity (%)	Density (${g/\mathrm{cm}}^{3}$)
Uncoated	55	1.6	3.15
CVD-0.50	48	3.3	3.01
CVD-1.00	46	6.9	2.84

**Table 5:** Properties of Tyranno^TM^-SA grade-3 polycrystalline SiC fiber, SiC matrix, and PyC interface.

Material Property	SiC Fiber [59,61]	SiC Matrix [62]	PyC Interface [63,64]
Young’s modulus E	409 GPa	400 GPa	30 GPa
Poisson’s ratio υ	0.185	0.2	0.4
Critical energy release rate $G_{c}$	${17.823\;J/m}^{2}$	${20\;J/m}^{2}$	$2.5{\;J/m}^{2}$
Fracture strength $\sigma_{\max}$	2510 MPa	700 MPa	119 MPa

**Table 6:** The real mechanical properties of SiC_f_/SiC_m_ composites under different interface conditions and the predicted flexural strength by numerical simulation.

	Real PyC Thickness (nm)	Simulation PyC Thickness (nm)	Experimental Flexural Strength (MPa)	Simulation Flexural Strength (MPa)	The Relative Error Compared to the Central Value of the Experiment (%)
Uncoated	0	0	1145±50	1040.38	9.14
CVD-0.50	100–250	500	600±26	460.81	23.20
CVD-1.00	450–600	1000	409±60	359.31	12.15

## Data Availability

The original contributions presented in this study are included in the article. Further inquiries can be directed to the corresponding author.
